# Molecular Mechanisms of Syndromic Cryptorchidism: Data Synthesis of 50 Studies and Visualization of Gene-Disease Network

**DOI:** 10.3389/fendo.2018.00425

**Published:** 2018-07-26

**Authors:** Kristian Urh, Živa Kolenc, Maj Hrovat, Luka Svet, Peter Dovč, Tanja Kunej

**Affiliations:** Department of Animal Science, Biotechnical Faculty, University of Ljubljana, Ljubljana, Slovenia

**Keywords:** candidate genes, comorbidity, cryptorchidism, diseasome, syndrome, undescended testes, systems biology, biological network

## Abstract

**Background:** Cryptorchidism is one of the most frequent congenital birth defects in male children and is present in 2–4% of full-term male births. It has several possible health effects including reduced fertility, increased risk for testicular neoplasia, testicular torsion, and psychological consequences. Cryptorchidism is often diagnosed as comorbid; copresent with other diseases. It is also present in clinical picture of several syndromes. However, this field has not been systematically studied. The aim of the present study was to catalog published cases of syndromes which include cryptorchidism in the clinical picture and associated genomic information.

**Methods:** The literature was extracted from Public/Publisher MEDLINE and Web of Science databases, using the keywords including: syndrome, cryptorchidism, undescended testes, loci, and gene. The obtained data was organized in a table according to the previously proposed standardized data format. The results of the study were visually represented using Gephi and karyotype view.

**Results:** Fifty publications had sufficient data for analysis. Literature analysis resulted in 60 genomic loci, associated with 44 syndromes that have cryptorchidism in clinical picture. Genomic loci included 38 protein-coding genes and 22 structural variations containing microdeletions and microduplications. Loci, associated with syndromic cryptorchidism are located on 16 chromosomes. Visualization of retrieved data is presented in a gene-disease network.

**Conclusions:** The study is ongoing and further studies will be needed to develop a complete catalog with the data from upcoming publications. Additional studies will also be needed for revealing of molecular mechanisms associated with syndromic cryptorchidism and revealing complete diseasome network.

## Introduction

Cryptorchidism or undescended testes is characterized as the failure of one (unilateral) or both (bilateral) testes and associated structures to descend from retroperitoneal abdomen to their normal position in scrotal sac during fetal development (1). It is one of the most frequent congenital birth defects in male children and is present in 2–4% of full-term male births (2). It has also been reported that cryptorchidism may develop after infancy, in some cases as late as young adulthood, but that is exceptional and was observed in only about 1–3% of male children (3). Around four out of five cases of cryptorchid testes descend by the first year of life (the majority within first 3 months) on their own (4).

Men with a history of cryptorchidism have an increased risk of infertility. Outcomes related to infertility include the paternity rate, semen analysis, measurement of serum luteinizing hormone (LH), follicle-stimulating hormone and inhibin B, and testicular size (5). Paternity rates in formerly bilaterally criptorchid men who have attempted to father a child (65.3%) are significantly lower than formerly unilaterally criptorchid men (89.7%) and control men (93.2%). Sperm density and inhibin B levels are lower in the bilateral group whereas follicle-stimulating hormone and luteinizing hormone levels are higher, compared to unilateral and control groups (6).

Hormone treatment with androgens, human chorionic gonadotropin (hCG) and luteinizing hormone-releasing hormone (LHRH), intended to sufficiently bring testes to a scrotal position, has so far proved to have limited success (7). However, it has been observed that in combination with orchidopexy (a surgical procedure to move undescended testes to scrotum), hormonal treatment significantly increased testosterone levels and the number of adult spermatogonia compared to orchidopexy alone (8). In all cases, orchidopexy in early stages of life is universally recommended since it prevents degeneration of spermatogenic tissue and decrease of spermatogonia which occurs if the issue is not resolved (9).

The second common effect of cryptorchidism is increased risk for testicular neoplasia, specifically seminoma and testicular torsion. About 2–3% of males born with unilateral or bilateral cryptorchidism develop testicular cancer, the risk factor is therefore ~4–40 times increased (1). Testes descent is a complex, multistage series of events requiring the interaction of several anatomical factors, including normal gubernaculum development, nervous system, and hormonal factors. Testes descent is described in three distinct phases: (1) the abdominal translocation; (2) followed by transinguinal migration; and (3) migration to the bottom of the scrotum. The developmental process, during which testes descend, was subdivided into three phases due to three general locations where non-scrotal testes were found (9).

In most cases, causes of cryptorchidism usually cannot be determined. It can occur as an isolated disorder in males with no other genital or birth defects, however, it can also appear as co-occurring disorder due to the presence of other congenital anomalies. It is viewed as a complex disorder which according to a high number of studies and research work in the recent years is believed to be direct consequence of combination of several factors such as: (1) environmental factors such as maternal health and chemicals acting as endocrine disruptors; (2) premature birth; (3) genetics causes such as polymorphisms in different genes and chromosomal aberrations; (4) comorbidity due to other abnormalities (2, 9).

Among the factors critically responsible for testicular descent are insulin-like factor 3 (INSL3) and hormones, in particular androgens such as testosterone. Mutations in genes encoding for these hormones or their receptors often lead to the occurrence of cryptorchidism. Insulin-like 3 is a peptide hormone produced by the Leydig cells and its expression is upregulated in the fetal testes and downregulated after birth. Deficiency of this hormone during the development of fetus can cause undescended testes. The role of insulin-like factor 3 is related to its effect on gubernaculum differentiation and development during the transabdominal phase (10). For androgens, it is generally believed that they act on both the cranial suspensory ligament and gubernaculum, normal functionality of the two is needed for regular descent of the testes since they are major mediators of the inguinoscrotal phase. During the transabdominal phase androgens also contribute to and aid the regression of cranial suspensory ligament. Mutations in androgen receptor (AR) gene are a frequent cause of cryptorchidism development. It has been observed that during development from fetus sex differentiation to puberty Sertoli cells produce anti-Müllerian hormone which prevents the development of the Müllerian ducts into the uterus and other Müllerian structures. It also enables normal development of Wolffian ducts, which eventually progress into male reproductive organs. However, the research in case of this particular hormone is still incomplete and inconclusive, therefore its role in cryptorchidism is still controversial (1).

Various genetic loci have been associated with cryptorchidism development, including protein-coding genes, chromosomal mutations, copy number variations and microRNAs (11). Genomic loci are dispersed through an entire genome, therefore a genome wide screening for identification of smallest regions of overlaps in cryptorchidism has been performed. These narrowed regions present candidate regions for further identification of stronger candidates and biomarker development (12).

Testicular maldescent can occur as an isolated event, or as part of a variety of syndromes (syndromic cryptorchidism) and other non-syndromic diseases (non-syndromic cryptorchidism) (13–15). Some syndromes with cryptorchidism in the clinical picture are extremely rare, with only few cases reported worldwide [for example 2p14p15 microdeletion syndrome (16)], while others are quite frequent [for example Beckwith-Wiedemann syndrome (1/13,000)] (1).

Molecular mechanisms for co-presence of symptoms in a syndrome could be explained using systems biology approach. For example, in our previous study molecular mechanisms underlying co-occurrence of cryptorchidism and cardiovascular diseases in RASopathies have been proposed (17). Foresta et al. reviewed syndromes that include cryptorchidism in clinical picture from OMIM database (1). Cryptorchidism gene database included 32 syndromes with cryptorchidism in clinical picture (11). However, the study field has not yet been systematically studied.

The aim of the present study was therefore to catalog the genetic loci associated with syndromic cryptorchidism. Genomic locations of candidate genes linked to development of syndromic cryptorchidism were visualized on a karyotype view to identify possible genomic hotspots associated with the development of cryptorchidism. The visualization of network consisting of syndromes connected to microdeletions or genes which caused them was also interpreted in systems biology manner as a contribution to growing diseasome map. The aim of the study is also to contribute to the standardization of reporting of congenital disorders and comorbidity defects together with corresponding hereditary causes.

## Materials and methods

Candidate genes associated with syndromic cryptorchidism were extracted from published literature as described previously (11). Various keywords were used for identification of syndromic cryptorchidism loci: syndrome, cryptorchidism, undescended testes, loci, microdeletions, and genes. Data from publications were obtained up to March 2018 and manually analyzed. Gene names were updated according to Ensembl genomic browser, release 92 (18). The network revealing connections between candidate genes and disorders was visualized using Gephi (19). Visualization of chromosomes and corresponding abnormalities associated with the development of cryptorchidism was performed using a figure of human karyotype downloaded from Ensembl genomic browser release 92 (18) and javascript programming language. Genomic location (base pairs) of breakpoints of chromosomal regions involved in chromosomal mutations were obtained from UCSC database (https://genome.ucsc.edu/cgi-bin/hgTables).

## Results

We retrieved data from PubMed and WoS. After manual elimination there were 50 studies left, from which we harvested data for current study. The catalog of genetic loci of syndromic cryptorchidism consists of 60 genetic loci associated with 44 syndromes that include cryptorchidism in the clinical picture. Among those 60 loci 38 were protein-coding genes and 22 of those loci were structural variations including microdeletions and microduplications. Among candidate genes residing within chromosomal locations the genes which were discussed or functionally analyzed to be associated with the development of a syndrome (or cryptorchid phenotype, if possible) in reviewed publications were cataloged and visualized in an interaction network.

### Catalog of genetic loci associated with syndromic cryptorchidism

Data were retrieved from PubMed and WoS. Study types of obtained publications including relevant genomic information were performed using different study approaches including: case reports, association, and functional studies, genome-wide and single locus studies, and different omics types. Syndromes that include cryptorchidism in clinical picture were reported to be associated with protein-coding genes and chromosomal mutations. Table 1 includes protein coding genes associated with syndromic cryptorchidism, which are alphabetically ordered by their locus names. Proteins, encoded by the genes listed include enzymes (BRCC3), hormones (AMH), transcription inhibitors (ANKRD11), inhibitors of enzymes (CDKN1C), transmembrane receptors (RET), and many other types and subtypes of regulatory proteins. Table 2 includes chromosomal mutations associated with syndromic cryptorchidism. Chromosomal mutations include microdeletions or microduplications, of various size ranging from 3.5 to 43.7 Mb. In some cases chromosomal mutations were associated with candidate genes, in total 8 possibly responsible for cryptorchidism phenotype. Each row in the catalog represents a genetic origin of a syndrome, containing gene or cytogenetic location of mutation, deletion or duplication, name of a syndrome, DOID (Disease ontology ID if available), reference of a publication in which the connection to cryptorchidism was proposed and PMID (PubMed ID) or OMIM ID. Chromosomal mutations are ordered by the chromosome number. The systematic approach enables future researchers to use this manually checked data in further studies more efficiently.

**Table 1:** Protein-coding genes, associated with syndromes with cryptorchidism in clinical picture.

**Locus name**	**Chromosome**	**Name of the syndrome**	**Disease ontology ID (DOID) for the syndrome**	**References**	**PMID (or OMIM ID)**
AMH	19	Persistent Müllerian duct syndrome (PMDS)	0050791	(20)	25026127
				(21)	28742509
ANKRD11	16	KBG syndrome	14780	(22)	21782149
ANOS1	X	Kallmann syndrome	3614	(23)	18160472
ATRX	X	Smith-Fineman-Myers syndrome	NA	(24)	10751095
BMP7	20	TDS syndrome	NA	(25)	22140272
BRAF	7	Cardiofaciocutaneous syndrome	60233	(26)	22190897
CDC6	17	Meier-Gorlin syndrome	0060306	(27)	22333897
CDKN1C	11	Beckwith-Wiedemann Syndrome	5572	(28)	20503313
CDT1	16	Meier-Gorlin syndrome	0060306	(27)	22333897
CHD7	8	CHARGE syndrome	0050834	(29)	17661815
				(30)	25606431
CHD7	8	Kallmann syndrome	3614	(31)	19021638
CHRM3	1	Prune Belly syndrome	0060889	(32)	22077972
EBP	X	MEND syndrome	NA	(33)	20949533
FGD1	X	Aarskog– Scott syndrome (ASS)	6683	(34)	23443263
GFRA1, RET	10	CAKUT	NA	(35)	22729463
HRAS	11	Costello syndrome	50469	(26)	22190897
				(36)	19213030
ICR1	11	Beckwith-Wiederman syndrome	5572	(37)	21863054
IRF6	1	Popliteal pterygium syndrome	0060055	OMIM (38)	119500
					12219090
KRAS	12	Noonan syndrome	3490	(39)	16474405
MAP2K2	19	Cardiofaciocutaneous syndrome	0060233	(40)	23885229
MBTPS2	X	BRESEK/BRESHECK	NA	(41)	22105905
MED12	X	Opitz-Kaveggia syndrome (OKS) (FC syndrome)	14711	(42)	17334363
MEGF8	19	Carpenter syndrome	0060234	(43)	23063620
NOTCH2	1	Hajdu-Cheney syndrome(HJCYS)	2736	(44)	25696021
NSD1	5	Sotos syndrome	14748	(45)	15942875
OCRL	X	Lowe oculocerebrorenal syndrome (OCRL)	1056	(46)	24778696
ORC1	1	Meier-Gorlin syndrome	0060306	(27)	22333897
ORC6	16	Meier-Gorlin syndrome	0060306	(27)	22333897
POLD1	19	Mandibular hypoplasia, deafness, progeroid features, lipodystrophy syndrome (MDP syndrome)	NA	(47)	23770608
PROK2	3	Kallmann syndrome	3614	(48)	18559922
PROKR2	20	Kallmann syndrome	3614	(48)	18559922
PTPN11	12	Noonan syndrome	3490	(26)	22190897
PTPN11	12	LEOPARD syndrome	14291	(49)	18505544
RAB23	6	Carpenter syndrome	0060234	(50)	20358613
RAB3GAP2	1	Martsolf syndrome	NA	(51)	16532399
RAF1	3	Noonan syndrome	3490	(40)	23885229
RAF1	3	LEOPARD syndrome	14291	(49)	18505544
RIT1	1	Noonan syndrome	3490	(52)	25124994
SEMA3A	7	Kallmann syndrome	3614	(53)	22416012
SOS1	2	Noonan syndrome	3490	(26)	22190897
TGFBR3	1	TDS syndrome	NA	(25)	22140272

*NA, not available*.

**Table 2:** Chromosomal regions, associated with syndromes with cryptorchidism in clinical picture.

**Chromosome**	**Locus name; chromosomal region and proposed candidate genes**	**Name of the syndrome**	**Disease ontology ID (DOID) for the syndrome**	**References**	**PMID (or OMIM ID)**
1; 10	NOTCH2; 46,XY,del(10)(q25.3-q26.13)	Persistent Müllerian duct syndrome (PMDS) with distal monosomy 10q	0050791	(54)	25820398
1	1p22–p21	Zellweger syndrome	905	(2)	15136137
2	del(2p14p15)	2p14p15 microdeletion syndrome	NA	(16)	23266801
2; X	46,XY,del(2)(p15p16.1); del(X)(q28), BRCC3^***^	2p15p16.1 microdeletion syndrome	0060415	(55)	22406401
3	3p22–p26	Fanconi anemia	13636	(2)	15136137
7	46,XY,dup(7p21)	7p22.1 microduplication syndrome	NA	(56)	21998864
				(57)	25124455
				(58)	25893121
8	46,XY,del(8)(q23q24)pat	Langer-Giedion syndrome (LGS) or Trichorhinophalangeal syndrome (TRPS) type II	4998	(59)	26269715
10	del(10q25.3q26.3), WDR11^*^,PLPP4^*^(PPAPDC1A)	10q deletion syndrome	NA	(60)	14598339
				(61)	18661548
				(62)	19253379
				(63)	19558528
				(64)	26114870
11	11p13	Denys–Drash syndrome (DDS)	3764	(2)	15136137
11	11p13	Genitourinary dysplasia- component of WAGR	NA	(2)	15136137
15	del(15q25.2), RPS17	15q25 deletion syndrome	0060396	(65)	20921022
15	15q11q13	Prader–Willi syndrome	11983	(2)	15136137
16	16p13	Rubinstein–Taybi syndrome	1933	(2)	15136137
17	del(17q21.31), KANSL1^**^	Koolen de Vries syndrome (KDVS)	0050880	(66)	18628315
				(67)	26306646
17	dup(17p12)	Oesophageal atresia/tracheoesophageal fistula and anal atresia	0080171	(68)	24239950
X	Xp21.1	Aarskog– Scott syndrome (ASS)	6683	(2)	15136137
X	Xq25–q27	Dandy–Walker syndrome (DWS)	2785	(2)	15136137
X	Xq28	Frontometaphyseal dysplasia	NA	(2)	15136137
X	(Xq27q28),ANOP1	Lenz dysplasia	NA	(2)	15136137
X	(Xp11.4p21.2), BCOR(ANOP2)	Lenz dysplasia	NA	(2)	15136137
X	Xq26.1	Lowe oculocerebrorenal syndrome (OCRL)	1056	(2)	15136137
X	Xq12–q21.31	Opitz-Kaveggia syndrome (OKS) (FG syndrome)	14711	(2)	15136137
X	Xq28	Torticollis, keloids, renal dysplasia, cryptorchidism (TKCR)	NA	(2)	15136137

*NA, not available*.

* *genes were determined via shortest region of overlap*.

** *a SNP in KANSL1 or del(17q21.31) is sufficient to cause KDVS*.

*** *the only gene on del(X)(q28) with an intragenic loss (4 exons)*.

### Genetic distribution of loci associated with syndromic cryptorchidism

**Figure 1:**
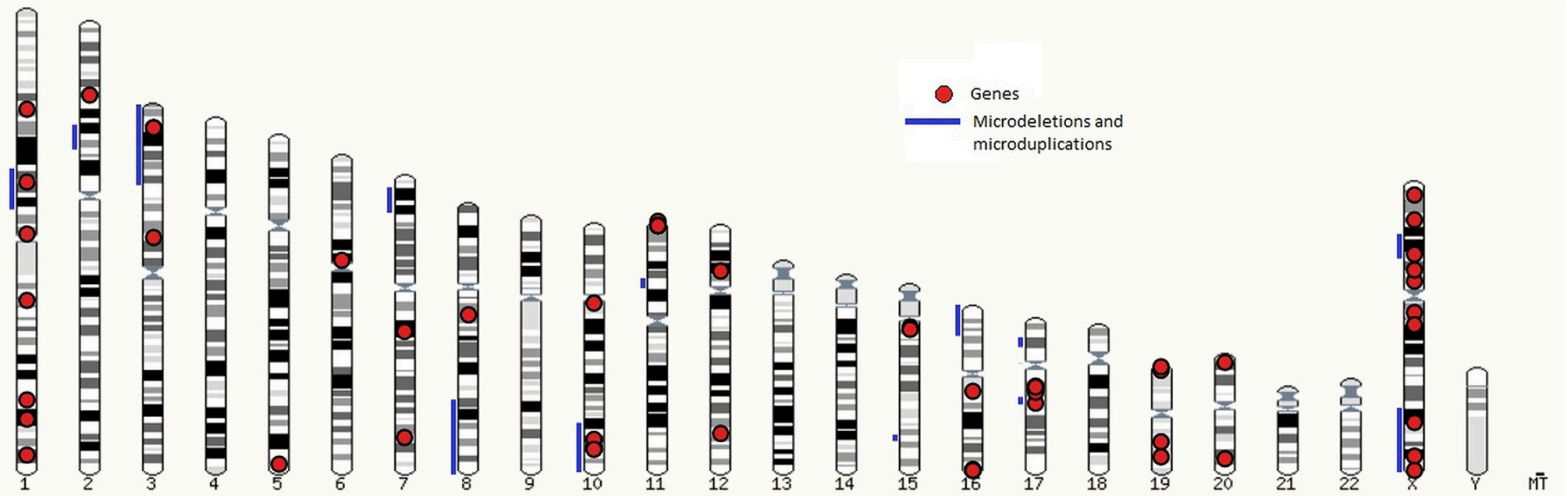
A karyotype view with loci associated with syndromic cryptorchidism in human.

Genomic location of loci linked to syndromes are presented in the karyogram view in Figure 1 which shows microdeletions and microduplications with blue lines and genes with red dots. Loci, associated with syndromic cryptorchidism are located on 16 chromosomes. Number of loci, associated with syndromic cryptorchidism per chromosome are shown in Supplementary Table 1. The highest number of loci, associated with syndromic cryptorchidism are located on chromosome X (*n* = 16) (for example Lenz dysplasia, X-linked Kallmann syndrome, and Aarskog-Scott syndrome) and chromosome 1 (*n* = 8) (for example Noonan syndrome, TDS syndrome, and Meier-Gorlin syndrome. Visualization of loci on a karyotype view revealed regions of overlaps, where multiple microdeletions coincide on one chromosome to produce single phenotype output, or causative genes located within chromosomal mutation. Analysis revealed that candidate gene *BRCC3* coincides with genetic loci recognized as a causative for TKCR syndrome. *BRCC3* gene is also considered causative for 2p15p16.1 microdeletion syndrome's cryptorchidism as there was a male case studied which, apart from microdeletion on chromosome 2, also had deletion on X chromosome in q28 region (55). Furthermore, the gene is also recognized as causative for MOYAMOYA disease which has wide range of testicular abnormalities in clinical picture (74).

As can be observed in Table 1, there are many different genetic causes associated with one syndrome. In Carpenter syndrome, for instance, there are two genes, *MEGF8* located on chromosome 19 (43), and *RAB23* located on chromosome 6 (50), malfunctions of each of them was associated with cryptorchidism. Interestingly, for Kallmann syndrome, five different genes were found to cause cryptorchidism, each of which is located on separate chromosomes: *PROK2* on chromosome 3 (48), *SEMA3A* on chromosome 7 (53), *CHD7* on chromosome 8 (31), *PROKR2* on chromosome 20 (48), and *ANOS1* on X chromosome (23). Such diversity is difficult to notice when data are scattered across different references, but come to attention when summarized in one analysis.

### Visualization of a gene-disease network view

**Figure 2:**
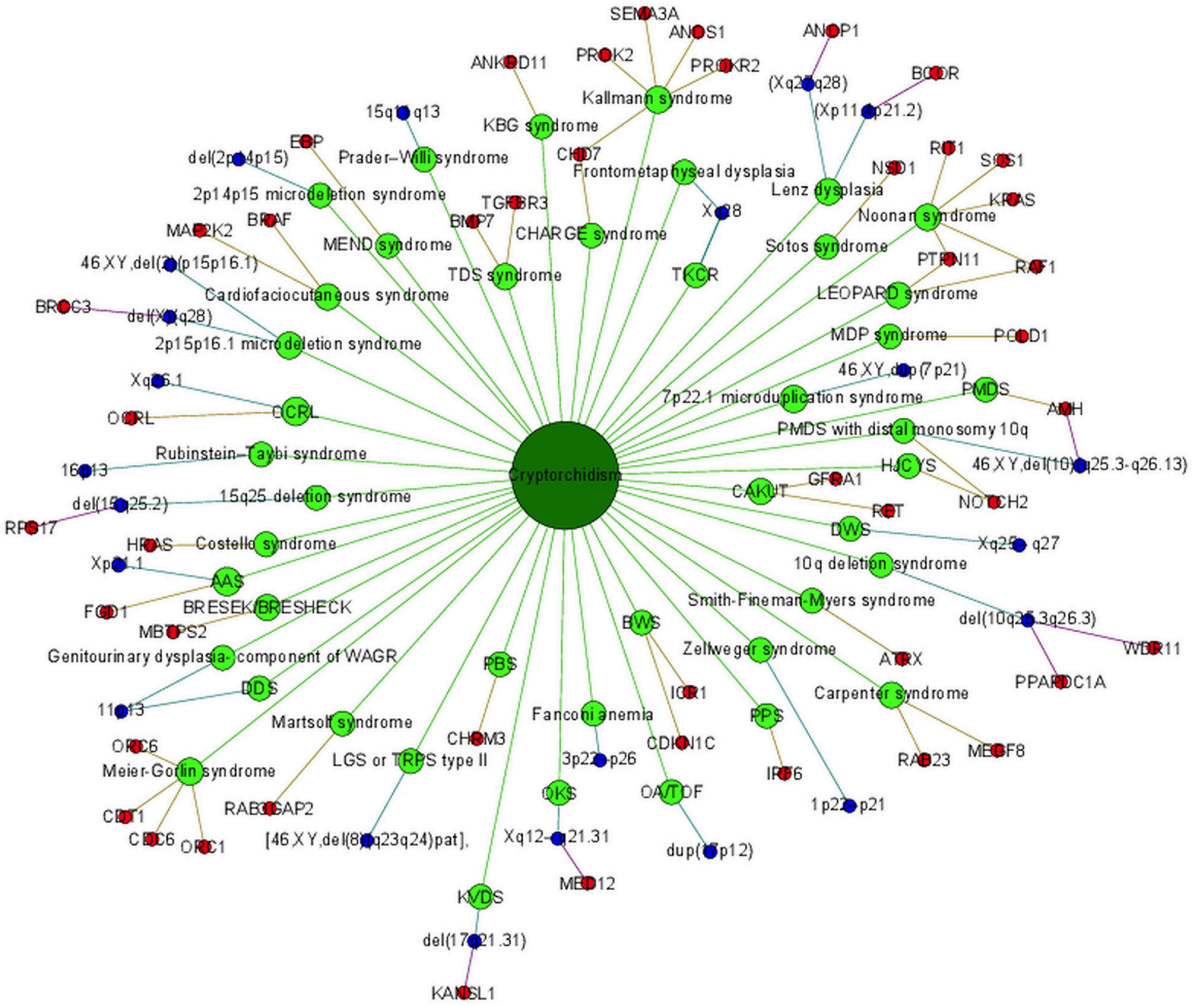
Portrayal of cryptorchidism associated syndromes and genetic loci.

Cryptorchidism often appears together with several other diseases and disorders or it is present in clinical picture of syndromes. Syndromes associated with cryptorchidism are presented in Figure 2. Figure 2 presents all 44 syndromes associated with cryptorchidism connected to 60 candidate genetic loci which include 38 genes and 22 chromosomal mutations. Some studies reporting chromosomal mutations associated with syndromic cryptorchidism additionally performed functional analysis or discussed one or more candidate genes potentially causing the syndrome. From those studies we extracted 8 genes which were proposed to contribute to development of cryptorchidism. The Figure 2 therefore includes 22 chromosomal mutations and 46 (38 + 8) genes associated with syndromes. Visualization presents the presence of cryptorchidism in several syndromes such as KBG syndrome, Kallmann syndrome, Noonan syndrome and Lenz dysplasia. Altogether there are 11 syndromes which are connected to at least one other syndrome via eight different genetic locations. For example, LEOPARD syndrome and Noonan syndrome, belonging to RASopathies, share several phenotypic features: characteristic facies, congenital heart defects, delayed development, and cryptorchidism for which the molecular causes are heterozygous mutations in various exons of *RAF1* and *PTPN11* (26, 40, 49). Likewise, as reported in a study by (31) features of Kallmann syndrome, hypogonadotrophic hypogonadism and anosmia or hyposmia are often present in patients with CHARGE syndrome for which the molecular cause is a mutation in *CHD7* as can be seen in Figure 2. Figure also revealed a great heterogeneity of associations in the molecular syndromology field. For example, a locus can be associated with: (1) a syndrome with cryptorchidism in the clinical picture, (2) can be a genetic cause for the development of a syndrome, but the patient does not have cryptorchidism in the clinical picture, or (3) a locus can be associated with another disease, which is not a syndrome. Examples of such heterogeneity of genotype-phenotype are presented in Table 3 (2, 23, 31, 48, 53).

**Table 3:** Example of heterogeneity of genotype-phenotype relations in association with cryptorchidism.

**Locus**	**Disease**	**Cryptorchidism present**	**References**	**PMID**
*SEMA3A*	Kallmann syndrome	yes	(53)	22416012
*ANOS1*	Kallmann syndrome	yes	(23)	18160472
*CHD7*	Kallmann syndrome	yes	(31)	19021638
*CHD7*	Kallmann syndrome	no	(31)	19021638
*CHD7*	CHARGE syndrome	yes	(31)	25606431
*PROK2*	Kallmann syndrome	yes	(48)	18559922
*PROKR2*	Kallmann syndrome	yes	(48)	18559922
Xp22.31	Kallmann syndrome	yes	(2)	15136137

## Discussion

The etiology of cryptorchidism is complex and has yet to be fully determined. Current evidence indicates that there are several factors that cause the disease, among which the most important ones are genetic and environmental factors (9, 17). If these elements significantly deviate from the ones seen in unaffected males, there is a high probability that cryptorchidism will appear. The results of research and literature mining have led us to conclusion that there is a vast quantity of seemingly unrelated syndromes co-occurring with cryptorchidism which are just being discovered and that specialized databases for syndromic cryptorchidism, do not exist yet.

Retrieving information of relevant genes from chromosomal mutations regarding cryptorchidism is a challenging task as propositions of candidate genes are presented in various ways. Some publications only listed a chromosomal location [for example 11p13 in Denys-Drash syndrome (2). Choucair et al. (64)] determined candidate *WDR11* and *PLPP4* (previous symbol *PPAPDC1A*) genes for genital anomalies development in patients with 10q deletion syndrome by smallest regions of overlap. The largest chromosomal mutation is present in Fanconi anemia, which extends over 43.7 Mb (2) and according to the latest version of the genomic browser Ensembl it includes 213 protein-coding genes. A study by Wat et al. (65) considered a role of *RPS17* (ribosomal protein S17) in 15q25.2 deletion syndrome. According to Ensembl genomic browser, this region extends is 3.5 Mb and includes 26 protein-coding genes. Within some microdeletion syndromes where candidate genes were not discussed causative genes for the occurrence of the syndrome can be found in OMIM database. For example, Prader-Willi syndrome in a study by Klonisch et al. (2) did not include candidate genes. Further investigation of OMIM database revealed that *NDN* and *SNRPN* are listed as candidate genes for Prader-Willi syndrome and that cryptorchidism is present frequently in the clinical picture of this syndrome. However, our study only includes cases in which genetic loci were associated with a syndrome and a patient also had diagnosed cryptorchidism. Such scattered information makes it very challenging to perform effective scientific research and the vast heterogeneity of genotype-phenotype relation which is why a standardized database of gene-disease interactions is necessary.

Investigation of comorbidity of diseases and genetic associations, as well as developing networks of disorders such as the Diseasome (69), allows elucidation of genotype-phenotype associations between several diseases that may have common genetic origin. The relevance of protein-protein interactions was examined in a study by Cannistraci et al. (17), where such interactions revealed the relation between co-presence of cardiomyopathy and cryptorchidism; two symptoms of the RASopathies. The study by Pevec et al. (70) also provided a baseline for future studies of associations between interactome and phenome in RASopathies, additionally presenting data at genome, interactome, and phenome levels and as an integrated network of all three data types.

Malfunctioning genes may result in seemingly unconnected phenotypes in different tissues which could hide the actual molecular background of such disease. Clustering on the basis of phenotypic similarities represents true biological relationships of the genes involved—we may then use such similarities to prioritize potential disease genes and make predictions of a certain phenotype (71). The visualizations presented in the current study could be seen as a tool for better and more perceivable understanding of connection between genotype and phenotype. Some complex interaction between genotype and phenotype have been published, for example, the TKCR syndrome (Torticollis, keloids, cryptorchidism, and renal dysplasia), which is caused by deletion of Xq28 (72) coincides with genomic coordinates of a *BRCC3* gene. Protein product of this gene is a part of a holoenzyme complex BRCC, which has a role in DNA repair mechanisms (73). This gene has been also mutated in a patient which had two diagnosed chromosomal mutations; 2p15p16.1 and Xq28 (55). Furthermore, *BRCC3* gene is also connected to MOYAMOYA disease (74) which also has similar symptoms including short stature, hypergonadotropic hypogonadism, facial dysmorphism, and in some cases decreased testicular volume and azoospermia (75). There are few candidates, for instance NOTCH2 and KANSL1, which play an important role in regulatory pathways, expressed in testicular cells. NOTCH2 is a membrane receptor protein with extracellular domain and an intracellular domain, which both play an important role in controlling cell fate decision pathways (76). Similarly, KANSL1 is a nuclear protein which has an important role in chromatin modifications as it is a part of a multiprotein complex that works as a histone acetylase (77). Both appear to be essential for proper cell differentiation during testicular development, although there has been no reported case of cryptorchidism caused just by mutation in *NOTCH2* gene, but it's contribution to reported, more complex cases, cannot be excluded (54). Additionally, *RPS17* encodes a protein subunit of ribosome, which is recognized as causative for Diamond-Blackfan anemia, but it has frequently coincided with syndromic cryptorchidism as well (65). A visualization tool simplifies search for causing genes and contributes to our molecular understanding of microdeletion syndromes. Our approach leads the integration of syndromic phenotypical abnormalities in the field of systems biology.

There are other syndromes which, according to a study by Foresta et al. (1) and OMIM database have cryptorchidism in the clinical picture, and also loci, where causative genes are not yet identified. That is why there is still possibility for further investigation that might lead us to understanding of novel signaling pathways involved in human development. As data is limited to few specific cases it is crucial that one extrapolates and includes new knowledge in systems of known data and therefore slowly unveil full picture behind complex genetic complications in human diseases. Various clinical subtypes of cryptorchidism exist, including unilateral, bilateral, syndromic, non-syndromic, comorbid, thus further studies are required to additionally clarify the role each particular gene contributes to different cryptorchid phenotypes. We must also not neglect the contribution of post transcriptional modification and epigenetic modification such as methylation and acetylation to development of different types of cryptorchidism.

As shown in Table 2 based on the collected data there are genomic hotspots on chromosomes X and 1 which have the highest number of genes associated with syndromic cryptorchidism. With knowledge of causative genes and other genetic loci one might develop a genetic therapy or hormonal treatment that would counteract the malfunctioning gene (78). Since the majority of genes are positioned on the X chromosome, genetic tests could be developed to consider applying treatment in advance of pregnancy and therefore counteract potential development of monogenic syndrome or at least minimize the symptoms.

The results of the present study contribute to the development of molecular syndromology field and understanding of associations between genome and phenome—Diseasome. The developed database and results of this study also contribute to better understanding of the genetic causes of cryptorchidism and associated comorbid syndromes. The developed protocol could now be also applied to other multifactorial traits and diseases. Further studies of the linkage between genetic variations and phenotype could contribute to the development of specific markers, which would in turn enable the diagnosis of the disease early during fetal development. Thus, better understanding of the causes for the disorder could also help to elucidate the complete mechanism that occurs during the development of testicular tissue—a process that is currently not completely understood.

## Executive summary

- Cryptorchidism is one of the most common genital defects in men which causes infertility, testicular neoplasia, psychological damage, and other health defects.
- Cryptorchidism is often comorbid with other symptoms and syndromes associated with chromosomal mutations and genetic variations.
- We have extracted data from WoS and PubMed and cataloged 60 genetic variants reported to be associated with syndromic cryptorchidism.
- Variants associated with syndromic cryptorchidism are heterogeneous and include 38 protein-coding genes and 22 structural variants (deletions, microdeletions, and microduplications). Using systems biology approach we visualized a biological network of genes connected to syndromes which present a baseline for further identification of novel candidate genes for cryptorchidism.
- Research methods and network visualization used in present study could be further extended to develop a complete diseasome and lead to systemic understanding of gene-phenotype relationship.
- The progress of the project was hampered by heterogeneous data presentation in published reports, therefore we call for standardization of gene and phenotype terminology in future publications. Independent validation in a larger number of cases will also be needed.

## Author contributions

KU, MH, ŽK and LS gathered data, conducted data analysis, and drafted the manuscript, MH and ŽK conducted graphical representation. TK designed and coordinated the study, PD and TK revised the final version of the manuscript.

### Conflict of interest statement

The authors declare that the research was conducted in the absence of any commercial or financial relationships that could be construed as a potential conflict of interest.

## Supplementary material

Supplementary Table 1 The number of genetic loci associated with syndromic cryptorchidism according to chromosome location.

## Abbreviations

KDVS: Koolen de Vries syndrome
DWS: Dandy-Walker syndrome
OKS: Opitz-Kaveggia syndrome (FC syndrome)
DDS: Denys-Drash syndrome
AAS: Aarskog-Scott syndrome
TKCR: Torticollis, keloids, cryptorchidism and renal dysplasia
OCRL: Lowe oculocerebrorenal syndrome
TDS: Testicular dysgenesis syndrome
MEND syndrome: Male EBP disorder with neurologic defects
HJCYS: Hajdu Cheney syndrome
PMDS: Persistant mullerian duct syndrome
BRESEK/BRESHECK: Brain anomalies, retardation, hirschsprung disease, ear/eye anomalies, cleft palate/criptorchidism and kidney dysplasia/hypoplasia
PPS: Popliteal pterygium syndrome
BWS: Beckwith-Wiedemann syndrome
LEOPARD syndrome: multiple Lentigines, ECG conduction abnormalities, Ocular hypertelorism, Pulmonic stenosis, Abnormal genitalia, Retardation of growth and sensorineural Deafness
PBS: prune belly syndrome
MDP syndrome: Mandibular hypoplasia, deafness, progeroid features, lipodystrophy syndrome
OA/TOF: Oesophageal atresia/tracheoesophageal fistula and anal atresia.
