# Supplementary material for: Molecular Mechanisms of Syndromic Cryptorchidism: Data Synthesis of 50 Studies and Visualization of Gene-Disease Network
Source: Front Endocrinol (Lausanne). 2018 Jul 26;9:425. doi: 10.3389/fendo.2018.00425 (PMC6070605; doi:10.3389/fendo.2018.00425)
Supplement: Supplementary Table 1 — The number of genetic loci associated with syndromic cryptorchidism according to chromosome location. [file Table_1.docx]

Supplementary Material

**Molecular Mechanisms of Syndromic Cryptorchidism: Data Synthesis of 50 Studies and Visualization of Gene-Disease Network**

Kristian Urh, Živa Kolenc, Maj Hrovat, Luka Svet, Peter Dovč, Tanja Kunej^*^

*** Correspondence:** Tanja Kunej, [tanja.kunej@bf.uni-lj.si](mailto:tanja.kunej@bf.uni-lj.si)

**Supplementary Table 1:** The number of genetic loci associated with syndromic cryptorchidism according to chromosome location.

| Chromosome | Number of loci |
| --- | --- |
| X | 16 |
| 1 | 8 |
| 11 | 4 |
| 16 | 4 |
| 19 | 4 |
| 10 | 3 |
| 3 | 3 |
| 7 | 3 |
| 17 | 3 |
| 2 | 2 |
| 8 | 2 |
| 12 | 2 |
| 15 | 2 |
| 20 | 2 |
| 5 | 1 |
| 6 | 1 |
| 4 | 0 |
| 9 | 0 |
| 13 | 0 |
| 14 | 0 |
| 18 | 0 |
| 21 | 0 |
| 22 | 0 |
| Y | 0 |
